# Sodium-DNA for Bone Tissue Regeneration: An Experimental Study in Rat Calvaria

**DOI:** 10.1155/2017/7320953

**Published:** 2017-09-10

**Authors:** Barbara Buffoli, Gaia Favero, Elisa Borsani, Ramon Boninsegna, Guido Sancassani, Mauro Labanca, Rita Rezzani, Pier Francesco Nocini, Massimo Albanese, Luigi Fabrizio Rodella

**Affiliations:** ^1^Section of Anatomy and Physiopathology, Department of Clinical and Experimental Sciences, University of Brescia, Brescia, Italy; ^2^Interdipartimental University Center of Research “Adaption and Regeneration of Tissues and Organs (ARTO)”, University of Brescia, Brescia, Italy; ^3^Centro Medico Duca d'Aosta, Brescia, Italy; ^4^Studio Dentistico Sancassani, Verona, Italy; ^5^Section of Oral and Maxillofacial Surgery, Department of Surgery, University of Verona, Verona, Italy

## Abstract

Surgical techniques in dental and maxillofacial surgery request fast bone tissue regeneration, so there is a significant need to improve therapy for bone regeneration. Several studies have recently underlined the importance of nucleotides and nucleosides to increase cell proliferation and activity; in particular, the ability of polydeoxyribonucleotide (PDRN) to induce growth and activity of human osteoblasts was demonstrated. Sodium-DNA is the deoxyribonucleic acid (DNA) extracted from the gonadic tissue of male sturgeon and then purified, depolymerized, and neutralized with sodium hydroxide. To date, there are no evidences about the use of Sodium-DNA for bone tissue regeneration. Consequently, our question is about the efficacy of Sodium-DNA in bone healing. For testing the role of Sodium-DNA in bone healing we used a rat calvarial defect model. Sodium-DNA at different concentrations used alone or in association with Fibrin and/or Bio-Oss was used for healing treatments and the bone healing process was evaluated by histomorphometric and immunohistochemical analyses. Our results suggested a positive effect of Sodium-DNA in bone regeneration, providing a useful protocol and a model for the future clinical evaluation of its osteogenic properties.

## 1. Introduction

Surgical techniques in dental and maxillofacial surgery request adequate and fast bone tissue regeneration. In recent decades, different surgical approaches have been proposed to manage different types of bone defects using a variety of graft materials with different osteoconductive, osteoinductive, and osteogenic properties. Nowadays, autologous bone graft and allograft are the most used devices [1–8], but the limited tissue availability and the risk of donor site morbidity (for autologous graft) and the possibility of host rejection or disease transmission (for allograft) represent some important limitations.

Several studies have underlined the importance of action of nucleotides and nucleosides to increase proliferation and activity of different cell types [9–12] by acting in synergy with several growth factors (i.e., epidermal growth factor, EGF, platelet-derived growth factor, PdGF, and fibroblast growth factor, FGF), modulating cytokines and growth factor production, and influencing immunological response [13].

Polydeoxyribonucleotide (PDRN) is a compound holding polymers of different length obtained from the sperm of some animal species and used as a tissue repair-stimulating agent. Its effects on cell growth and activity have been demonstrated in different cell lines [12–18] and tissues [18–27]. In particular, PDRN is cleaved by active cell membrane enzymes, providing a source for deoxyribonucleotides and deoxyribonucleosides that can increase cell proliferation and activity stimulating nucleic acid synthesis through the salvage pathway [28] and/or binding and activating the purinergic receptors [9, 20, 23]. Evidences suggested that PDRN, acting as an agonist on adenosine A_2A_ receptor, was able to improve healing process by increasing the expression of vascular endothelial growth factor (VEGF) and angiopoietin-1, an angiogenic factor involved in the stabilization and maturation of newly formed vessels [12, 20, 23, 25, 29]. Moreover, an important role in maintaining cell proliferation and in preventing the exaggerated hyperproliferation that may be associated with tissue repair has been also suggested [19].

In bone tissue regeneration, PDRN was reported to play an important role acting as osteoblast growth stimulator. In this way, purinergic receptors seem to be partially involved in the rapid proliferation, new bone formation, and a reduction of bone healing time, as confirmed after treatments with specific purinergic receptor inhibitors [9, 13, 18, 28]. PDRN effect on bone regeneration was also demonstrated in an experimental study on rats and mice, in which the effect of PDRN used alone or in association with other materials was reported [30, 31].

Sodium-DNA is the deoxyribonucleic acid (DNA) extracted from the gonadic tissue of male sturgeon and then purified, depolymerized, and neutralized with sodium hydroxide. Sodium-DNA passes through the cell membrane by pinocytosis and acts as a donor of purine and pyrimidine bases, which are key molecules for cell vitality. To date, there are some evidences about its efficacy in the treatment of skin lesions [32] but there are no literature data concerning its potential effects in dental and maxillofacial surgery.

Therefore, the aim of this study was to evaluate the efficacy of Sodium-DNA used alone or in association with Fibrin and/or Bio-Oss (Geistlich, Wolhusen, Switzerland), a bone substitute material obtained from the mineral portion of bovine bone, for repairing bone defect in a rat calvarial experimental model. In addition to histomorphometric evaluation, we examined, immunohistochemically, three markers of bone regeneration: RUNX2, an essential transcription factor for osteoblast differentiation and for extracellular matrix gene expression [33, 34]; osteocalcin (OCG3), a marker of osteocalcin, which is produced by osteoblasts and is implicated in bone mineralization and calcium ion homeostasis [35]; osteopontin (OPN), a phosphoglycoprotein of the extracellular matrix of bone tissue that plays an important role in bone resorption as is expressed by many other cells [36, 37].

## 2. Materials and Methods

### 2.1. Experimental Design

The experimental protocol was approved by the Local Ethical Committee on Animal Care and Use of the University of Brescia and by the Italian Ministry of Health. Sixty male Wistar rats (Harlan, Milan, Italy) weighing between 320 to 420 g each were used in this study. The animals were held in separate cages in a ventilated stand, under standardized air and light conditions at a constant temperature of 22°C with a 12-hour light/day cycle. They had free access to tap drinking water and standard laboratory food pellets.

The animals were randomly divided into 6 groups (10 animals for each group). Each group was subdivided in relation to the different time treatment in two subgroups (30 and 60 days) of 5 animals each. The animals of Group III, IV, V, and VI were treated differently for the right osteotomy (A) and the left osteotomy (B). Following, we reported experimental design.

*Group I*. Control: the defects were unfilled. 

*Group II*. Fibrin: the defects were filled with Fibrin Glue (Tisseel, Baxter AG, Vienna, Austria). 

*Group III (A-B)*. (A) Bio-Oss: the defects were filled with Bio-Oss (Geistlich Biomaterials, Wolhusen, Switzerland); (B) Fibrin + Bio-Oss: the defects were filled by a mixture of both. 

*Group IV (A-B)*. (A) Fibrin + DNA-Na (Sanaryn, Veritas srl, Brescia, Italy): the defects were filled by a mixture of both; (B) Fibrin + vehicle (glycerol, silanol mannuronate, and nisin) of DNA-Na: the defects were filled by a mixture of both. 

*Group V (A-B).* (A) Bio-Oss + DNA-Na: the defects were filled by a mixture of both; (B) Bio-Oss + vehicle of DNA-Na: the defects were filled by a mixture of both. 

*Group VI (A-B)*. (A) Fibrin + Bio-Oss + DNA-Na: the defects were filled by a mixture of all; (B) Fibrin + Bio-Oss + vehicle of DNA-Na: the defects were filled by a mixture of all.

### 2.2. Surgical Procedure

The animals were anesthetized with an intraperitoneal injection of Zoletil 100 (60 mg/kg body weight; Virbac, France) containing a mixture of tiletamine and zolazepam. The dorsal region of the skull was shaved and the head of the rat was positioned in a cephalostat during the operative procedure and aseptically prepared for surgery. A middle skin incision from the nasofrontal area to the external occipital protuberance was performed. The skin and underlying tissues, including the temporalis muscle, were reflected laterally to expose the full extent of the calvaria. The periosteum surrounding the defect was removed to prevent periosteum osteogenesis. Two symmetrical full-thickness 5 × 8 mm bone skull defects were made on each parietal region, lateral to the sagittal suture, using piezoelectric ultrasonic bone surgery under constant irrigation with sterile saline solution, which allows a selective cut of only mineralized structures without causing bone necrosis by heating [38]. The dura mater was preserved. This experimental surgical protocol represented a critical size defect, resulting in no spontaneous closure of the bone defect, not even reaching 50% of the area after 16 weeks of observation [39]. In order to minimize the number of animals used for this study, right and left osteotomy were made according to the experimental design. Furthermore, to ensure the same volume of test treatments, the defects were filled using stainless-steel syringes. At the end of the procedures, the flap of each animal was closed with silk sutures. After 30 or 60 days from surgery, the animals were sacrificed and the tissue within the original surgical defect area was removed. The tissue samples were fixed in 10% formalin, decalcified in Osteodec (Bio-Optica, Milan, Italy), and embedded in paraffin according to the standard procedures. Serial sections (7 *μ*m) were cut longitudinally by a microtome, starting from the center of the original surgical defect.

### 2.3. Histomorphometric Analysis

Histomorphometric analysis was performed to evaluate the percentage of new bone formation within the bone defect area. Sections were stained with Masson-Goldner Trichrome (Merck KGaA, Darmstadt, Germany). All sections were evaluated under an optical microscope (Olympus, Milan, Italy) by two investigators unaware of the group assignment. The following criteria were applied to standardize the histomorphometric analysis: (1) the total defect area was identified by the anterior and posterior margin of the surgical defect area and was delimited; (2) the area of newly formed bone was delineated within the selected total area.

Percentage of new bone (% NB) formation was calculated as area of newly formed bone expressed as percentage of the total defect area. Digitally fixed images were randomly analyzed using an image analyzer (Image Pro Premier 9.1; Immagini e Computer, Milan, Italy). The measurements were made as percentage of area in five sections for each sample.

### 2.4. Immunohistochemical Analysis

Tissue sections were processed for immunohistochemical analysis to detect RUNX2, osteocalcin (OCG3), and osteopontin (OPN).

Before the immunohistochemical assays, the sections were deparaffined, hydrated, and heat treated in 0.05 M EDTA buffer pH 8.0 (Bio-Optica, Milan, Italy) for antigen unmasking at 98°C for 20 minutes and RT for 20 minutes. Endogenous peroxidase activity was blocked by incubation with a solution of 3% hydrogen peroxide. Sections were immunostained with the following monoclonal antibodies: RUNX2 (mouse monoclonal, Abcam, Cambridge, UK); OCG3 (rabbit polyclonal, Abcam, Cambridge, UK); OPN (rabbit polyclonal, Abcam, Cambridge, UK). All sections were processed using UltraVision Quanto Detection System HRP (Thermo Scientific, Bio-Optica, Milan, Italy) and diaminobenzidine (Amresco, Prodotti Gianni, Milan, Italy). Section incubated without the primary antibody served as negative control.

Quantitative analysis of immunopositivity was performed to calculate the percentage area of immunostaining within the total defect area. The analysis was performed blindly, using an optical light microscope (Olympus, Germany). Digitally fixed images of slices were analyzed using an image analyzer (Image Pro-Plus 4.5.1; Immagini e Computer, Milan, Italy). The measurements were made as percentage of area in five sections for each sample (five random fields for section).

### 2.5. Statistical Analysis

Quantitative values of histomorphometric and immunohistochemical analysis were reported as mean ± standard error (SE). Statistical analysis was performed using one-way analysis of variance (ANOVA test) with Bonferroni correction. The significance level was set as *P* < 0.05.

## 3. Results

Experimental design also included treatments with vehicle of DNA-Na (Groups IVB, VB, and VIB) to exclude any of its possible effects on new bone formation. All these groups showed similar values with the groups treated with Fibrin alone (Group II), Bio-Oss alone (Group IIIA), or a combination of Fibrin and Bio-Oss (Group III-B). Therefore, we decided not to consider these groups in the data processing step of this study.

### 3.1. Histomorphometric Analysis

**Figure 1:**
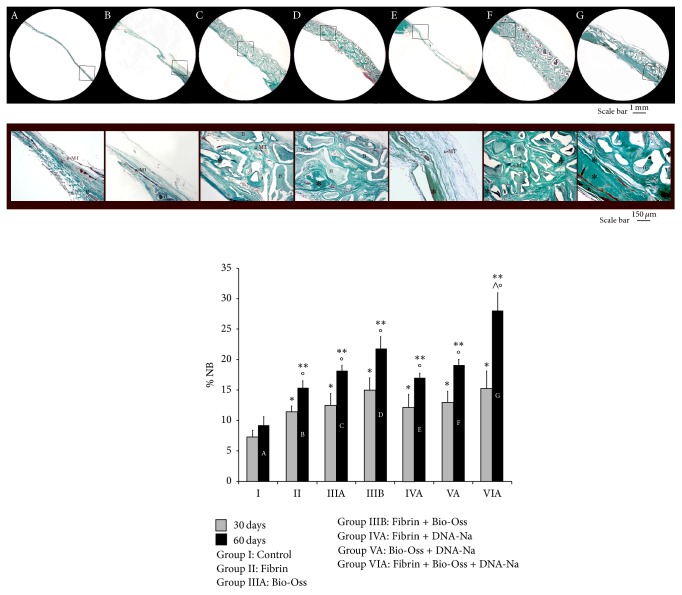
A–G: Masson-Goldner Trichrome at 60 days (up: low-magnification images; down: high-magnification images). ^*∗*^New bone. B: Bio-Oss; n-MT: nonmineralized tissue. Below: quantitative analysis of percentage of new bone formation (% NB) at 30 days (gray) and 60 days (black). ^*∗*^*P* < 0.05 versus Control (Group I) 30 days; ^*∗∗*^*P* < 0.05 versus Control (Group I) 60 days; °*P* < 0.05 versus 30 days into each group; ^∧^*P* < 0.05 versus all other groups.

Histomorphometric analysis was performed in order to quantify the percentage of new bone (% NB) formation within the total defect area. The results showed a significant increase after 60 days with respect to 30 days for all groups, excluding the Control group. Comparing the groups within the same time treatment (Groups II, III, IV, V, and VI), we observed a significant increase of % NB with respect to respective Control (Group I), after both 30 and 60 days from surgery. The significantly highest value of % NB was found in Fibrin + Bio-Oss + DNA-Na group after 60 days from surgery (Group VIA). Quantitative data were reported in Figure 1.

### 3.2. Immunohistochemical Analysis

Immunohistochemical analysis was used to investigate the immunolocalization of some markers of bone regeneration: RUNX2, osteocalcin (OCG3), and osteopontin (OPN). Negative control treated without the primary antibodies showed no positive staining (data not shown).

#### 3.2.1. RUNX2

**Figure 2:**
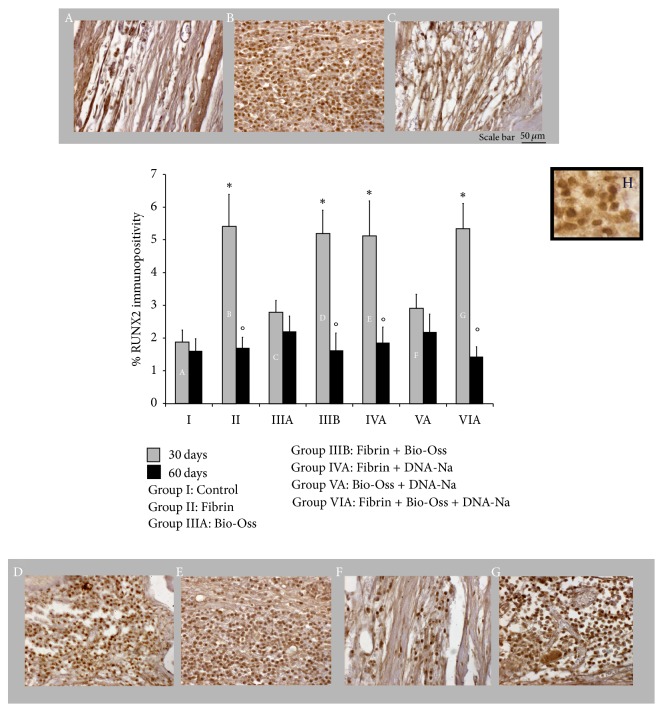
A–G: RUNX2 immunohistochemistry at 30 days. H: high resolution detail. Below: quantitative analysis of percentage of RUNX2 immunostaining at 30 days (gray) and 60 days (black). ^*∗*^*P* < 0.05 versus Control (Group I), Bio-Oss (Group IIIA), and Bio-Oss + DNA-Na (Group VA); °*P* < 0.05 versus 30 days into each group.

Immunohistochemical analysis of RUNX2 showed intracellular localization of immunostaining. No immunopositivity was observed in mature bone tissue. Quantitative analysis of immunostaining revealed a significant increase after 30 days in all groups treated with Fibrin (Groups II, IIIB, IVA, and VIA) respect to the others (Groups I, IIIA, and VA). A decrease in RUNX2 immunostaining was observed at 60 days with respect to 30 days for each group; this decrease was significant in the groups treated with Fibrin. No significant difference was observed at 60 days among all groups. Quantitative data were reported in Figure 2.

#### 3.2.2. OCG3

**Figure 3:**
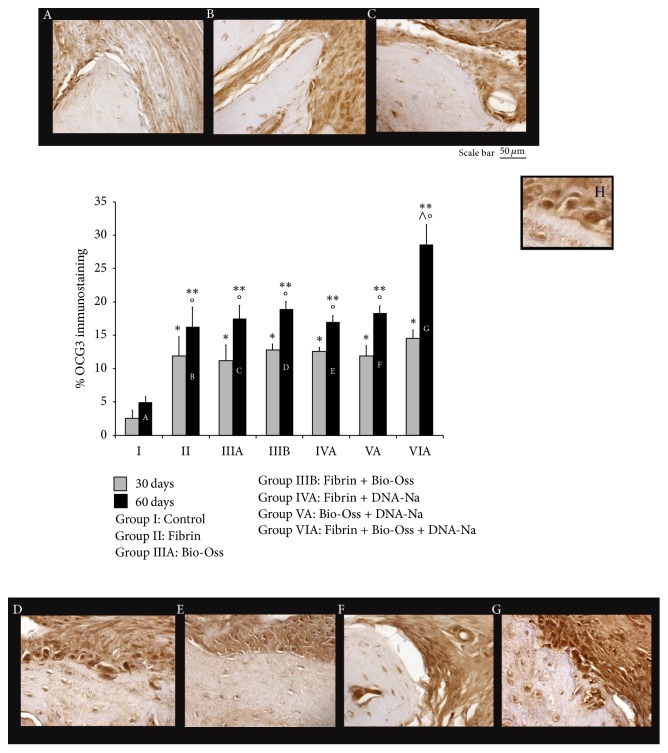
A–G: OCG3 immunohistochemistry at 60 days. H: high resolution detail. Below: quantitative analysis of percentage of OCG3 immunostaining at 30 days (gray) and 60 days (black). ^*∗∗*^*P* < 0.05 versus Control (Group I) 30 days; ^*∗∗*^*P* < 0.05 versus Control (Group I) 60 days; °*P* < 0.05 versus 30 days into each group; ^∧^*P* < 0.05 versus all other groups.

OCG3 immunostaining was localized in the osteoblasts that lied on the new bone surface and in the osteocyte lacunae. Quantitative evaluation showed a significant increase after 60 days from surgery, for all groups, excluding the control group. However, a significant increase was observed in all groups with respect to their Controls (Group I), after both 30 and 60 days from surgery. The highest value was found in Fibrin + Bio-Oss + DNA-Na group after 60 days from surgery (Group VIA). Quantitative data were reported in Figure 3.

#### 3.2.3. OPN

**Figure 4:**
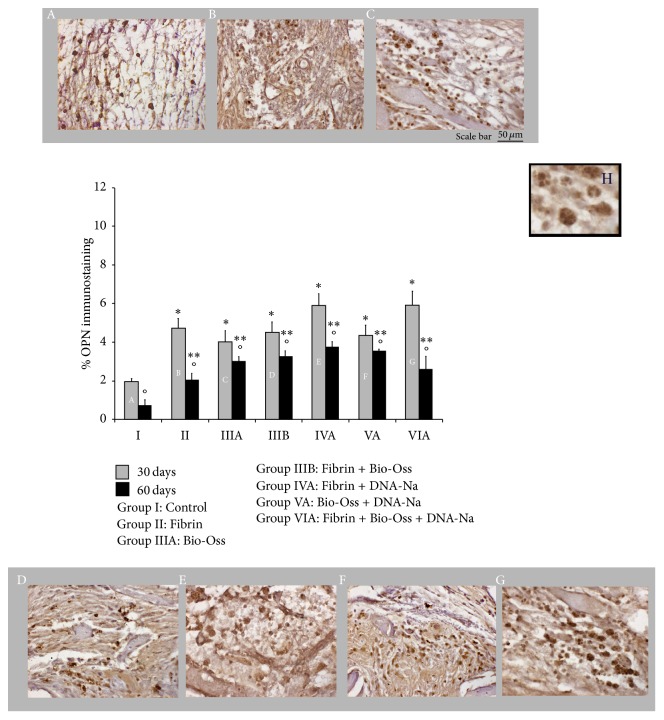
A–G: OPN immunohistochemistry at 30 days. H: high resolution detail. Below: quantitative analysis of percentage of OPN immunostaining at 30 days (gray) and 60 days (black). ^*∗∗*^*P* < 0.05 versus Control (Group I) 30 days; ^*∗∗*^*P* < 0.05 versus Control (Group I) 60 days; °*P* < 0.05 versus 30 days into each group.

Immunohistochemical analysis of OPN showed intracellular localization of immunostaining. A marked signal was observed within the cells scattered in nonmineralized tissue. Quantitative analysis showed a significant increase in all groups with respect to their controls, after both 30 and 60 days from surgery. However, significant highest values were found at 30 days and that came down to a significant decrease at 60 days in all groups, including the Control group (Group I). Quantitative data were reported in Figure 4.

## 4. Discussion

This study represents the largest in vivo study evaluating the effects of Sodium-DNA treatment in the context of bone regeneration. We used a rat calvarial defect model to evaluate, by histomorphometric and immunohistochemical point of view, bone healing effect of Sodium-DNA combined with or without Fibrin and/or Bio-Oss.

Our starting point was some literature evidences about the effects of PDRN on osteoblast proliferation and bone healing process [13, 30, 31]; in addition, there were some data about Sodium-DNA as skin repair active principle [32].

Our histomorphometric results showed a significant increase in bone regeneration using the combination of Fibrin + Bio-Oss + DNA-Na after 60 days from surgery. This could be explained considering the different properties for each material.

A “good material” for bone regeneration, in fact, should have osteoinductive, osteoconductive, and osteogenic properties at the same time [40, 41]: the combination among Fibrin, Bio-Oss, and DNA-Na could meet these characteristics.

Combining Fibrin glue and bone granules we had different benefits. First, it produced a mouldable material that was surgical handling, and then the Fibrin permitted cementing the granules into the implant site. However, in addition to the mechanical aspect of the composite, it was suggested that the combination might promote bone regeneration [42–45]. Consequently, Bio-Oss provided an osteoconductive site for the bone tissue growing and Fibrin represented the osteoinductive glue, according to the literature data reporting both the osteoconductivity of Bio-Oss [46, 47] and the effect of Fibrin sealant or Fibrin glue in osteoinductive process [42–45].

In addition, our data suggested that, among the three biologic mechanisms that provide a rationale for bone grafting, osteogenesis could be promoted by Sodium-DNA. This hypothesis was supported by other previous data about the effect of PDRN as osteoblast growth stimulator. In particular, these studies showed that PDRN action could be partially due to a stimulation of the purinergic system mediated by A_2_ purinoreceptors, suggesting its possible use as osteoblast stimulator for repairing bone defects [13, 28]. Moreover, PDRN effect on bone regeneration was also demonstrated in an experimental study on rats and mice, in which the effect of PDRN used alone or in association with other materials was reported [30, 31].

Furthermore, our immunohistochemical results gave us information about the activation of bone regenerative process. We investigated three markers: RUNX2, OCG3, and OPN.

RUNX2 is a transcription factor involved in osteoblastic differentiation and skeletal morphogenesis. It is essential for the osteoblast maturation and both intramembranous and endochondral ossification. It can directly stimulate transcription of osteoblast-related genes such as those encoding osteocalcin [33, 34]. RUNX2 is upregulated in immature osteoblasts, and then it is downregulated in mature osteoblasts [48]. Carroll and collaborators showed that purine (i.e., cAMP) was needed for full upregulation of RUNX2 and/or that finer tuning of cAMP levels is important for the Control of RUNX2 expression [49]. These studies supported our results; in fact, we found highest RUNX2 immunopositivity after 30 days from surgery, especially in Fibrin treated groups. This last point could be due to the osteoinductive property of the Fibrin that led to recruitment of the mesenchymal cells and to activation of RUNX2 and, consequently, to osteoblast differentiation.

OCG3 is a marker of osteocalcin. Osteocalcin is produced by osteoblasts during bone formation and it is implicated in bone mineralization and calcium ion homeostasis [35]. OCG3 immunostaining showed time-dependent increase in all experimental groups; the highest value was observed after 60 days in Fibrin + Bio-Oss + DNA-Na group. This result was in accordance with our histomorphometric data. Therefore, we could assume that Sodium-DNA, together with osteoinductive and osteoconductive properties of Fibrin and Bio-Oss, could improve osteogenesis.

OPN is a phosphoglycoprotein of the extracellular matrix of bone tissue that is also present in other different cells, such as macrophages, T lymphocytes, smooth muscle cells, and epithelial and ganglion cells [36, 37]. Our data showed significant higher values of OPN immunostaining after 30 days from surgery in all experimental groups; on the other hand, a significant decrease was observed after 60 days. Considering that OPN is necessary for bone remodeling and that it is suppressed when bone formation is stabilized, our supposition was that, during the earlier regenerative phases, OPN was expressed by granulation tissue (composed of fibroblasts, myofibroblasts, inflammatory cells, extracellular matrix, and newly formed vessels), as previously suggested [50].

## 5. Conclusion

These results suggested a positive effect of Sodium-DNA in bone regeneration, providing a useful protocol and a model for the future clinical evaluation of its osteogenic properties and an alternative method of therapy for patients suffering from bone defects that are both effective and economic.
